# Defining Standard Data Reporting in Pelvic Exenteration Surgery for Rectal Cancer: A PelvEx Collaborative Review of Current Data Reporting

**DOI:** 10.3390/cancers17172764

**Published:** 2025-08-25

**Authors:** PelvEx Collaborative

**Keywords:** pelvic exenteration, rectal cancer, outcomes, core outcome sets, locally advanced rectal cancer, locally recurrent rectal cancer

## Abstract

Introduction: Pelvic exenteration (PEx) is a radical procedure used in the treatment of locally advanced (LARC) and locally recurrent rectal cancer (LRRC). With recent advancements in perioperative treatment regimens, there has been renewed interest in this procedure as it offers the opportunity for complete tumour resection in a select cohort. This has resulted in large heterogeneity in outcome reporting, making comparing and conducting a meta-analysis of published results challenging. Standardising outcome reporting will ensure meaningful data reporting and allow the cross-centre comparison of data. Aims: To conduct a systematic review of the current literature, to identify the various outcomes reported for PEx in rectal cancer, and to develop a standard outcome reporting set. Methods: A systematic review was carried out following the PRISMA guidelines. Relevant domains were identified first. Data elements (DEs) were extracted verbatim prior to standardisation and mapping to relevant domains. Results: There has been a noticeable trend of increased literature on PEx in the last decade. Forty-nine papers were identified. A total of 1549 DEs were extracted verbatim. These were standardised to 119 unique DEs mapped to ten distinct domains capturing the patient care journey. There was large variation in the frequency of reporting, with some key outcomes reported in a limited number of studies. Conclusions: There is considerable heterogeneity at present in data reporting for PEx in LARC and LRRC. Standardisation of outcomes is the first step in guiding the development of a core information set to overcome heterogeneity and guide future research development.

## Simple Summary

Pelvic exenteration is a radical surgery for locally advanced and recurrent rectal tumors. Advances in perioperative care have renewed interest in this once palliative procedure, offering a potential cure for select patients. However, this renewed interest has corresponded with an influx in the volume of literature reporting on this procedure, which has led to an increase in outcomes reported by various authors. We aim to systematically review and document all outcomes currently reported in the field of pelvic exenteration for rectal cancer. This study is the initial step in a multi-step project aimed at creating a comprehensive core data set that includes core outcomes, measurements, and descriptors through a Delphi process involving all key stakeholders. The final core data set will help guide future data collection, analysis, and treatment strategies for this rare procedure. Additionally, this core data set will serve as the foundation for future PelvEx multi-center collaboration and will inform guideline development across our network.

## 1. Introduction

Rectal cancer management has continued to grow in complexity over the last decade. Locally advanced rectal cancer (LARC) and locally recurrent rectal cancer (LRRC) represent a specific subset of patients who have tumours extending beyond the TME plane. Complete resection remains the most important prognostic factor in the surgical treatment of these patients [1]. Since radial margins may be compromised due to malignant growth in proximity to or into adjacent structures, aggressive multi-visceral resection is often required to ensure complete disease resection. Pelvic exenteration (PEx) offers this solution as it aims to remove all pelvic organs (along with local bone or neurovascular involvement in select cases), offering locoregional control [2].

With the evolution in perioperative, anaesthetic care and advancements in chemotherapy and radiotherapy treatment strategies, increasing numbers of patients are becoming eligible to undergo PEx as survival trends increase [3,4]. Coupled with novel, minimally invasive approaches [5], and the feasibility of extended radical PEx to remove bone disease and metastectomy for select cases [6,7], PEx offers a potential “cure” for complete disease resection in a selective cohort [8]. Despite this, PEx still comes at a cost, as this radical surgery is associated with significant morbidity, irrespective of the technique [9]. The increasing amount of literature surrounding this topic has invariably resulted in large heterogeneity in outcome reporting, as there is no current consensus on which outcomes are clinically relevant; furthermore, no current guidelines define these outcomes [10].

The general complexity of PEx surgery, the significant morbidity that it carries, and the invariable impact on patients’ quality of life means that the quality assessment of key postoperative outcome measures is essential to ensure that patients receive the highest standard of care and allow clinicians to recognise early deviations from a standard perioperative course. This is usually achieved by reporting on key outcomes, which are clinically relevant results, such as the length of hospital stay, blood loss, etc. However, it is important that the outcomes reported are significant for the patient’s perioperative surgical journey and have a direct impact on their surgical course, disease, and quality of life. Standardisation of reporting and outcome definitions under a core information set (CIS) ensures that patients are receiving the highest standard of care, as it allows clinical centres to compare their current practices with international standards [11]. Furthermore, this allows for the easier analysis of reported data between various clinical institutes and the identification of current clinical shortcomings and facilitates meta-analyses of the current literature [12].

The identification of currently reported data elements (DEs) in the literature is an essential first step towards the development of a CIS in pelvic exenteration. Our aim was to systematically analyse the current literature, extract reported DEs, and standardise similar outcomes under one DE to pave the road for the development of a CIS.

## 2. Methods

The methodology of our review was guided by the Core Outcome Measures in Effectiveness Trials (COMET) Handbook [13] to guide development of a CIS. Our protocol, detailing the steps involved in achieving this, has been prospectively registered with the COMET initiative: “https://www.cometinitiative.org/Studies/Details/3212” (accessed on 1 March 2024). Our review, including the search strategy, study selection, and analysis, has been prospectively registered with the international Prospective Register of Systematic Reviews (PROSPERO Registration ID: 561629). The PRISMA reporting guidelines were employed in conducting our systematic review [14].

### 2.1. Search Strategy

An electronic search of PubMed/MEDLINE, Embase, Scopus, and the Cochrane Register of Controlled Trials was performed. Additionally, the obtained articles’ references were screened for relevant publications. A comprehensive search strategy using the following search terms in combination with Boolean AND/OR operators was used to generate our search string: “rectal cancer” OR “rectal neoplasm” or “rectal adenocarcinoma” OR “rectal malignancy” AND “pelvic exenteration” OR “multi visceral resection” OR “pelvectomy” OR “extended resection”. Our search was limited to papers published in the English language and published after the year 2000. After importing the collected articles to the Covidence software, all articles were screened by two reviewers before the appropriate abstracts were reviewed. The last search was performed on 10 April 2024.

### 2.2. Inclusion Criteria

Studies were to be included if they reported on PEx as an intervention for rectal cancer and primarily focused on outcomes of PEx. Included studies must have included at least ten study participants. Acceptable study designs included retrospective cohort studies, prospective cohort studies, randomised trials, cross-sectional studies, and qualitative studies.

### 2.3. Exclusion Criteria

Study designs excluded from our review included literature reviews, narrative reviews, conference abstracts, case reports, and cohort studies with ten patients or less. Studies reporting on exenteration for non-rectal cancer (except for colon cancer if it represented less than 10% of the study population) were also excluded. Studies that included intended cytoreductive surgery along with PEx were also excluded, along with those on patients who underwent concomitant surgical procedures besides PEx +/− sacrectomy +/− simple metastectomy. Studies reporting on palliative PEx procedures were also excluded.

### 2.4. Data Extraction

Two authors (M.M.S., É.J.R.) independently screened titles and abstracts for eligibility using the Covidence review software. A third reviewer resolved any disagreements (M.E.K.). The same reviewers independently screened full-text articles as required to determine eligibility. All screening was performed independently, and, at each stage, disagreement (as indicated by Covidence) was resolved by discussion until a consensus was reached. Following the completion of the paper selection process, a data charting form using Microsoft Excel (Microsoft, Redmond, Washington, DC, USA) was jointly developed by the two reviewers to collect standardised outcomes from the final papers.

### 2.5. Study Quality/Bias Assessment

Potential bias within our included non-randomised trials was assessed using the ROBINS-I tool [15]. This assessment tool graded each study as being of low risk (green circle), high risk (red circle), or unclear risk (yellow circle).

### 2.6. Data Cataloguing

Given the large heterogeneity among outcomes, we used reflexive thematic analysis [16] to create standardised outcomes combining verbatim data elements of similar concepts or capturing similar data—for example, flap sepsis, flap ischemia, and flap necrosis were captured as flap failure under our reconstructive domain. Standard terms such as this were chosen on the basis of capturing all similarly reported outcomes while still sufficiently specific to reflect that outcome. Outcomes were extracted and catalogued on Microsoft Excel (Microsoft, Redmond, Washington, DC, USA) under standardised headings if already included and as a newly agreed upon standard outcome if previously not captured. Multiple advisory meetings were held throughout the duration of this study with several senior consultant surgeons with experience relating to exenteration surgery to agree on standardised outcomes to ensure the appropriate categorisation of the data. This cataloguing of data and the standardisation process solely related to the terminology used moving forward. Variations in definitions were noted in various studies relating to several key data elements; however, defining individual outcomes fell outside the scope of this study. Our outcomes were categorised under one of ten domains. These domains are based on those originally proposed by the Core Outcome Measures in Effectiveness Trials [17] initiative but have been modified to suit the objectives of our study.

## 3. Results

**Figure 1:**
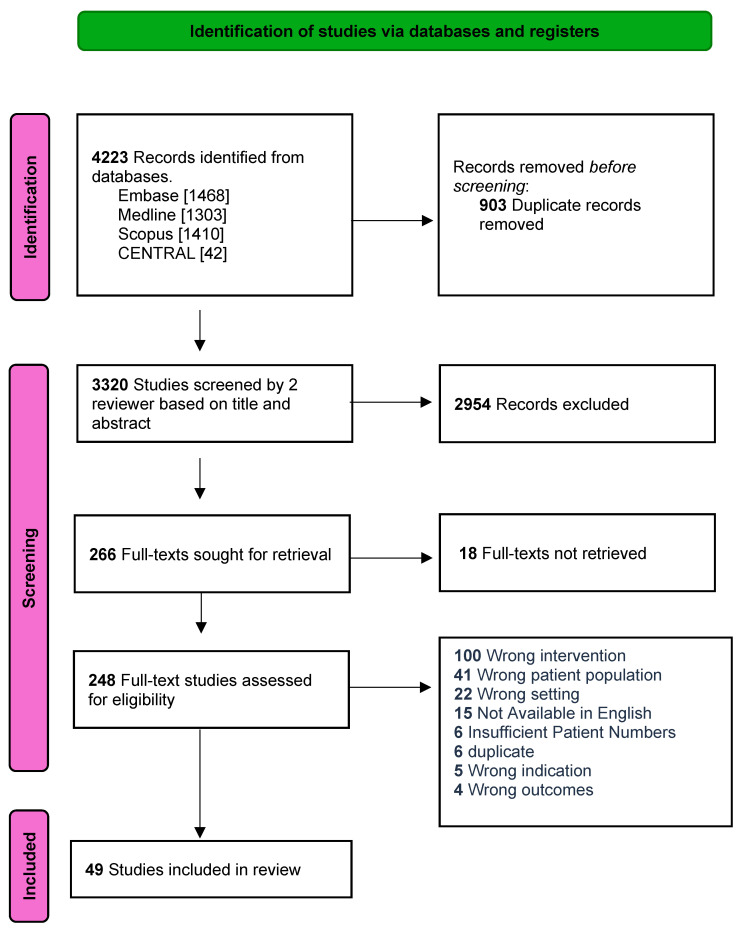
Flow diagram (using a PRISMA template) outlining the progression of studies throughout the review process.

Our search identified a total of 4223 studies, of which there were 903 duplicates. In total, 3220 studies were screened, and 266 papers were sought for full-text retrieval, of which 49 studies met our inclusion criteria [18,19,20,21,22,23,24,25,26,27,28,29,30,31,32,33,34,35,36,37,38,39,40,41,42,43,44,45,46,47,48,49,50,51,52,53,54,55,56,57,58,59,60,61,62,63,64,65,66] (Figure 1). These included one retrospective registry review, twelve prospective observational, and 36 retrospective observational studies.

The included literature shows a trend of an increased volume of papers reporting on the field of pelvic exenteration in rectal cancer in the last decade, with 81.6% of our included studies published from 2011 onwards (Table 1). Furthermore, it shows that there is also a variation in geographical publication on this technique, with Australia and Asia accounting for 49% of all published papers, followed by Europe with 26.5%. The most common study designs identified were retrospective observational cohort studies, which accounted for 73.5% of all studies. The 49 studies included a total of 12,164 patients (range: 11–2472 patients).

### 3.1. Data Reporting

**Figure 2:**
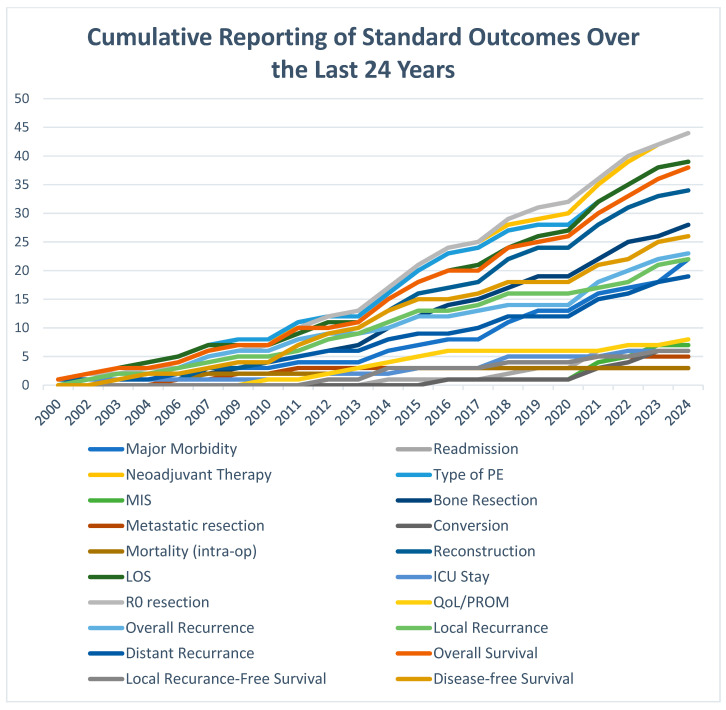
Cumulative reporting of standard outcomes over the last 24 years. A thematic reflection of outcome reporting in individual studies as standardised outcomes proposed in this study. The graph reflects an increase in the number of studies reporting on each of the selected outcomes over the study period.

**Figure 3:**
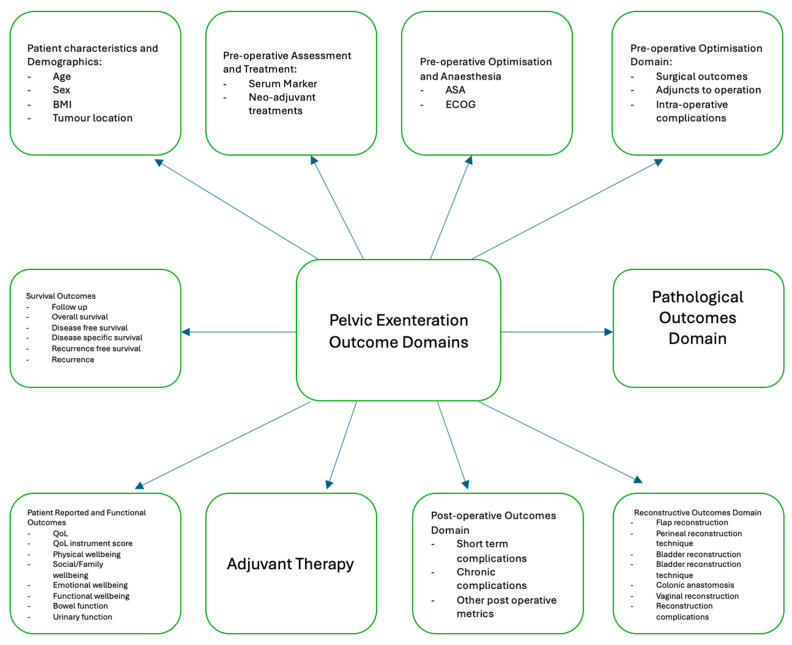
Mapping of standardised outcomes to domains.

A total of 1549 DEs were extracted verbatim from our included studies. These were standardised to 119 unique DEs (Table 2). Cumulative reporting of individual DEs revealed an overall trend of increased DE reporting as the years progressed (Figure 2). Our final standard DEs were mapped to ten broad domains (Figure 3), which were “Patient Characteristics/Demographics”, “Neoadjuvant Treatment”, “Preoperative Assessment and Anaesthetic”, “Intraoperative/Surgical Management”, “Pathological Outcomes”, “Reconstructive Outcomes”, “Postoperative”, “Adjuvant Therapy”, “Patient-Reported and Functional Outcomes”, and “Survival Outcomes”.

### 3.2. Study Bias

**Figure 4:**
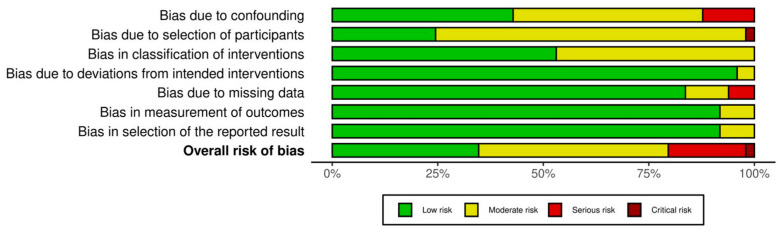
Risk of bias associated with observational studies (*n* = 49 observational studies).

The risk of bias associated with observational studies is outlined in Figure 4. Ten studies were identified as being seriously or critically biased [18,24,26,27,33,40,43,50,60,62]. The domains associated with the highest risk of bias, in descending order, were confounding factors, bias due to missing data, and bias due to the selection of participants. However, this did not impact the quality of our study, as the aim was to only extract reported outcomes and not analyse the reported data.

### 3.3. Patient Characteristics/Demographics

Patient demographics included outcomes that were the most commonly reported across all domains. These included patient age in 46 papers (93.88%) and sex in 44 (89.8%). Two other outcomes were included this domain, namely BMI and tumour location within the rectum, but these had lower levels of reporting, at 32.65% and 20.41%, respectively. Tumour location within the rectum, as a standard outcome, encompassed papers that reported the location as “upper vs. middle vs. lower rectum”, as well as papers that reported on the “tumour distance from anal verge” (Table 3).

### 3.4. Neoadjuvant Treatment

DEs relating to some form of neoadjuvant management were reported in 42 papers. Individual standard outcomes included unspecified neoadjuvant treatment in nine papers (18.37%) and neoadjuvant chemotherapy only in 20 studies (40.82%). Of note, reference to neoadjuvant consolidation chemotherapy was noted in two papers (4.08%) and total neoadjuvant therapy in two papers (4.08%). The chemotherapy drug regimen was highlighted in five papers (10.2%). Other outcomes under this domain included short-dose chemoradiotherapy in five papers (10.2%), long-dose chemoradiotherapy in four papers (8.16%), radiotherapy +/− chemotherapy in 32 papers (65.31%), and reference to the radiotherapy dose in 11 studies (22.45%). Only six studies (12.24%) reported on the treatment response to neoadjuvant therapy (Table 3).

### 3.5. Preoperative Assessment and Anaesthetic

Two subdomains were included under this heading, including a preoperative anaesthetic and serum marker subdomain. Two standardised DEs were mapped to the preoperative anaesthetic subdomain. These were ASA status and ECOG status. ASA status was reported in 17 studies (34.69%), while ECOG status was only reported in one study. The individual study that reported on ECOG status was also noted to report on ASA status [36] (Table 3). Serum markers reported preoperatively included CEA, albumin, haemoglobin, and Ca-19-9, which were reported in six, five, four, and one papers, respectively.

### 3.6. Intraoperative/Surgical

Given the large span of the intraoperative/surgical domain, three subdomains to categorise standard DEs were formulated under this heading. These were “surgical outcomes”, “adjuncts to operation”, and “intraoperative complications”. Surgical outcomes included 12 unique outcomes, which were reported in varying frequencies. However, there were several key outcomes that were well reported consistently across the literature, which included the number of resected compartments in 39 (79.59%), operative time in 36 (73.47%), and blood loss in 31 studies (63.27%). A minimally invasive surgical approach was outlined in seven studies (14.29%). Outcomes in relation to conversion to open (including papers where reference was made to zero conversions) were included in six of these seven studies. Other outcomes under this subdomain included references to bone resection at the time of PE in 27 studies (55.1%), the use of a foreign material to stabilise the bone following resection in one study (2.04%), metastatic disease (non-bone) resection in five studies, the volume of intraoperative transfusion in ten papers, and operative mortality in three papers. One individual paper reported on urinary output and fluid balance intraoperatively [58], both of which were included as unique standard outcomes. Two DEs were reported under our “adjuncts” subdomain. These were intraoperative radiotherapy in eight studies (16.33%) and hyperthermic intraperitoneal chemotherapy in one study (2.04%). Four DEs were extracted under intraoperative complications. These were major blood loss in five studies (10.2%), sciatic nerve injury and iatrogenic bowel injury in two studies each (4.08%), and ventricular fibrillation in one study (Table 3).

### 3.7. Pathological Outcomes

Pathological DEs were among the best-reported outcomes across the included papers and included R0 resection referenced in 43 studies (87.76%), R1 resection in 42 (85.71%), R2 resection in 23 (46.94%), and lateral surgical margin/circumferential resection margin in six studies (12.24%). Seven studies outlined the definitions used for R0 and R1 resection. Six of these studies had the same definition, referencing R0 as microscopically clear margins of at least 1 mm and R1 tumours as having margins involved microscopically if within 1 mm. One study, however, defined R0 as “clear microscopically” and R1 as “involved microscopically” [33], with no reference to margins. The tumour stage, grade, and size were also reported in 28, 13, and 6 studies, respectively. While the overall stage was reported for the majority of patients, some authors reported individual TNM components, but these were defined under one standard outcome of overall stage. Other DEs included lymphovascular invasion in eleven papers (22.45%), perineural invasion in five papers (10.2%), tumour regression grade in two papers (4.08%), and pathological necrosis and quality of mesorectum in one paper (2.04%) (Table 3).

### 3.8. Reconstructive Outcomes

Six unique DEs were mapped to this domain. This included flap reconstruction in 28 papers (5714). Only 13 of these papers, however, reported on the flap reconstruction technique that was employed. Five papers reported on flap complications. Flap complications as a standard outcome combined verbatim outcomes reporting on “flap breakdown”, “flap necrosis”, or “refashioning of perineal flap”. Bladder reconstruction was reported in 20 papers (40.82%), with 13 papers highlighting the reconstructive options that were employed. Urinary conduit complications were another standard outcome that combined verbatim outcomes relating to conduit stenosis, stricture, abscess formation, or unclassified conduit complication but strictly not anastomotic leak. Anastomotic leak was captured as a unique standard outcome reported in 24 papers (48.98%), which included both leaks from the conduit as well as bowel. Necrosis of the ileal conduit was another unique outcome reported in one paper. The last two remaining outcomes under the reconstructive domain were vaginal reconstruction in three papers (6.12%) and colonic anastomosis in 12 studies (24.49%) (Table 3).

### 3.9. Postoperative Outcomes

This was a broad domain covering postoperative complications as well as outcomes relating to postoperative return to function. Complications were divided into acute and chronic complications, as defined by the paper. Complications were assumed to be acute unless specifically outlined to be chronic in the individual study. There was a wide variety of acute and chronic complications, with frequencies of reporting given in Table 3. These standardised outcomes included major complications (as defined by each individual study), infection (unspecified), foreign device infection, wound complications (unspecified), wound infection, wound dehiscence, hernia formation, stoma complications, urinary infection, urinary retention/incontinence, ureteric leak, ureteric stricture, ileus, bowel obstruction, bowel perforation, intra-abdominal/pelvic sepsis, osteomyelitis, fistula formation, cardiac complications, respiratory infection, respiratory failure, DVT/pulmonary embolism, liver failure, renal complications, neurological complications, multiorgan failure, vocal cord paresis, pressure sores, bleeding, reoperation, and readmission. Only four chronic complications were identified. These included chronic pain in one, neuropathic bladder in two, urethral obstruction in one, and recurrent UTIs in two studies. Other outcomes reported under this domain included hospital stay, reported in 39 studies (79.59%), ICU stay in six papers (12.24%), time for bowel activity to return in four papers (8.16%), need for TPN in two papers (4.08%), and postoperative mortality within 90 days in 32 studies (65.31%). Adjuvant therapy, namely adjuvant chemotherapy +/− radiotherapy, was the only DE mapped to this domain, with references in 17 studies (34.69%).

### 3.10. Functional and Patient-Reported Outcomes

This was the least reported domain overall, with only nine unique papers referencing at least one DE under this domain. Standardised functional outcomes included quality of life (QOL) impact in six studies, the QOL instrument score in four studies, physical wellbeing in four studies, and emotional wellbeing in two studies. One paper each reported on of social/family wellbeing, functional wellbeing, bowel function, and urinary function (Table 3). The QOL tools utilised included the FACT-C, the SF36v2, and the AQOL scale.

### 3.11. Survival Outcomes

Survival domain DEs included the follow-up duration in 27 (55.1%), disease recurrence in 23 (46.94%), local recurrence in 21 (42.86%), and metastatic recurrence in 19 studies (38.78%). Outcomes highlighting patient survival included overall survival in 37 (75.51%), disease-free survival in 25 (51.02%), local disease-free survival in six (12.24%), and metastatic disease-free survival and disease-specific survival in one study each (2.04%) (Table 3).

## 4. Discussion

PEx is a highly complex procedure associated with significant morbidity. Comprehensive assessment, optimisation, and patient selection are essential to ensure that patients obtain the best survival outcomes and quality of life and prevent further tumour recurrence. Furthermore, the rise in minimally invasive PEx along with selective compartment PEx means that further meticulous patient selection to match each patient with the most appropriate approach is essential to avoid unwarranted complications and ensure complete disease resection. Studies reporting on PEx should reflect all components of this journey, from the clinical decision-making stage to postoperative survival. The main finding of the present study is that there is large heterogeneity in which outcomes are reported, with reporting standards not reflective of the complexity of this process.

As expected, age and sex were the most commonly reported features (93.88% and 89.8% of studies, respectively), followed by “classical” surgical outcomes [67] including the number of pelvic compartments removed, operative time, and blood loss (79.59%, 73.47%, and 63.27%, respectively). In contrast, certain demographic elements that can significantly impact the surgical process and postoperative recovery, such as BMI, were only reported in 32.65% of studies analysed. ASA grade is another important preoperative factor but was only reported in 34.69% of studies. This highlights some of the gaps in the literature currently around standard outcome reporting, as key DEs that have the potential to greatly impact the perioperative course are being overlooked. Other well-reported outcomes related to key pathological measures, especially in relation to complete resection (R0), which was reported in 87.76% of studies, were remaining microscopic disease (R1), reported in 85.71%, and macroscopic disease (R2), which was reported in 46.94%. This is of particular importance as clear margins are the most important predictor of long-term survival and quality of life [64] and thus a key benchmark measure of the success of these procedures. Bone resection where needed is another factor that has been identified to be a key prognostic indicator of overall survival and was reported in 55.1% of cases [64]. However, the definition of these pathological DEs was missing in most cases, with only 22.44% of studies reporting a definition. Furthermore, we found two major varying definitions regarding margin positivity, with ten papers defining R0 as microscopically clear within 1 mm (i.e., no tumour on ink) and R1 as having microscopic disease or involvement within this 1 mm margin. One study, however, reported R0 as clear microscopically and R1 as involved microscopically, without defining any margins required [33].

Postoperative complications were among the most well-documented postoperative outcomes. However, the majority of papers reported these as individual complications, which makes the comparison of such outcomes across multiple studies difficult. Twelve studies reported clinical outcomes using the Clavien–Dindo (CD) classification [68]—either for all complications or to define major complications in the case of six of these papers. Although individual outcome reporting is important to be able to identify the spectrum of normal postoperative complications, the lack of use of a classification system means that the severity of these complications cannot be quantified and compared between studies. The use of a system such as the CD framework or the more novel Comprehensive Complication Index [69] allows the quantification of the impacts of such complications and any necessary interventions on patients and their postoperative course, facilitating an analysis across the literature.

As previously outlined, PEx is a morbid procedure, with a trade-off in certain aspects relating to quality of life being unavoidable. As such, patients must be at the centre of the discussion process early on. Previous patient experience and functional impact is an essential measure, as outcomes that are important for clinicians may exhibit an unacceptable trade-off for patients. As such, the influence of this literature is significantly limited by the relative rarity of reporting of patient and functional-related outcomes. Patient outcomes such as urinary and bowel function were both only reported in one included study. This highlights the greater emphasis in the literature at present on clinical outcomes over patient-reported outcomes. The instrument used to assess quality of life appeared consistent across the board, with the FACT-C used in five studies, the SF36v2 used in four studies, and the AQOL used in one study. Nonetheless, studies that did report on quality of life used two instruments in 83.3% of cases, which helped to capture a wider spectrum of postoperative patient performance.

Heterogeneity in outcome reporting has been a longstanding issue in the field of colorectal cancer [70]. This has been previously addressed in the broader field of colorectal cancer by the development of a CIS for colorectal cancer surgery [71]. Heterogeneity in the field of PEx has been previously demonstrated by Brown et al. [72]. They have also demonstrated a recent trend of increased publications relating to pelvic exenteration, with large variation in outcome reporting and definitions pertaining to these. PEx has only emerged recently as the gold standard for LARC and LRRC [73]. This may explain why, at present, there is no consensus on which outcomes are important to report, as well as the variations in definitions being employed by various authors. However, as this procedure increases the opportunities for the development of a robust CIS, it is becoming more critical than ever before.

Our study has several strengths. Firstly, our search strategy and selection criteria narrowed the focus of this review solely to rectal cancer patients undergoing PEx. We also selected studies that exhibited an intention to cure rather than a palliative approach. By selecting such studies, the key outcomes extracted could help to guide future efforts in managing patients with “curable LARC and LRRC” by developing a CIS. We hope to achieve this by next identifying a consensus among key stakeholders, including patients, regarding which outcomes are essential to include, as well as identifying any gaps in the current literature. This will guide a final Delphi process to develop a final CIS, as described in our previously published protocol. However, we do acknowledge some limitations. Due to the study selection process, our review only pertains to rectal cancer and so cannot be generalised to all PEx. Furthermore, due to the search being limited to studies after the year 2000 and only including studies available in English, as well as the exclusion of certain study types including grey literature, further outcomes may have been extracted. However, given the descriptive nature of this study, the inclusion of non-English studies may not have been fully feasible given the need to understand the terminology used thoroughly, which may have been lost in translation if using translational services. Furthermore, given the number of studies included, it is unlikely that any new DEs not previously captured would have been extracted from these studies. We also acknowledge that it may not be practical for all future studies to comment on all outcomes that we describe. Studies reporting on other existing databases, such as the American NCDB, will lack some of the outcomes highlighted here. Nonetheless, having a standard set of outcomes will help to raise the level of reporting in some databases.

In conclusion, there is significant heterogeneity in outcome reporting related to PEx for LARC and LRRC. At present, there is no consensus on which outcomes should be reported. This study underscores the necessity of standardised outcome reporting in PEx. The significant variability in reported outcomes, across broad domains such as demographics, surgical metrics, and postoperative complications, highlights gaps that impact the assessment of patient care outcomes and research comparability. Emphasis should be placed on ensuring the comprehensive reporting of both clinical and patient-centred outcomes, including quality of life and functional impacts, to truly reflect the complexity of PEx. Establishing a robust CIS is essential to unify reporting standards, ensuring that all relevant outcomes are consistently documented. This review is the first regarding the standardisation process for a CIS. By providing a gross outline of all currently reported outcomes in the field of PEx, it will form the basis of planned focus group surveys aimed at identifying irrelevant data elements and key missing outcomes and defining these terms to help guide a future Delphi process.

## 5. Conclusions

Our review identified 119 DEs across ten domains, which various studies currently report on in the field of PEx in rectal cancer. By categorising and standardising the DEs currently being reported, this will form the basis of our focus group questionnaires aimed at guiding a Delphi process to standardise outcome reporting in PEx. Our goal is a complete data set capturing patients’ preoperative, operative, and postoperative statuses to facilitate future data collection across the PelvEx network and guide evidence synthesis and benchmarking across all involved centres. This will foster enhanced patient management and help to improve research in the field of exenterative surgery in the future.

## Figures and Tables

**Table 1:** Study characteristics (*n* = 49).

Characteristic	Number (%)
**Publication Period:**	
2000–2005	4 (8.2)
2006–2010	5 (10.2)
2011–2015	13 (26.5)
2016–2020	13 (26.5)
2021–2024	14 (28.6)
**Geographical Distribution:**	
Nort America	6 (12.2)
South America	1 (2)
Europe	13 (26.5)
Asia	17 (34.7)
Australia and New Zealand	7 (14.3)
Middle East	1 (2)
International Multicentre	4 (8.2)
**Study Type**	
Retrospective Observational	36 (73.5)
Prospective Observational	12 (24.5)
Retrospective Registry Review	1 (2)

**Table 2:** Domains and associated data elements (*n* = 119).

Domain	Number of Outcomes Identified
Patient Characteristics/Demographics	4
Preoperative assessment and Treatment	14
Preoperative Optimisation/Anaesthetic	2
Intraoperative/Surgical Management	18
Pathological Outcomes	13
Reconstructive Outcomes	10
Postoperative Outcomes	40
Adjuvant Therapy	1
Patient-Reported and Functional Outcomes	8
Survival Outcomes	9

**Table 3:** PEx standardised domains and data elements.

Domain	Outcome	Number (%)
Patient Characteristics and Demographics	Age	46 (93.88)
	Sex	44 (89.80)
	BMI	16 (32.65)
	Tumour location within rectum (upper vs. middle vs. lower)/distance from anal verge	10 (20.41)

Neoadjuvant Treatment	Neoadjuvant treatment (unspecified)	9 (18.37)
	Neoadjuvant chemotherapy only	20 (40.82)
	Neoadjuvant chemo regimen used	5 (10.2)
	Neoadjuvant consolidation chemo	2 (4.08)
	Total neoadjuvant therapy	2 (4.08)
	Neoadjuvant radiotherapy +/− chemo	32 (65.31)
	Short-dose CRT/CxT	5 (10.2)
	Long-dose CRT/CxT	4 (8.16)
	Radiotherapy dose	11 (22.45)
	Response to neoadjuvant treatment	6 (12.24)

Preoperative Assessment and Anaesthetic	*Serum Markers*	
	CEA	6 (12.24)
	Albumin level preop	5 (10.2)
	Hb level preop	4 (8.16)
	CA-19 9	1 (2.04)

	*Preoperative Anaesthetic*	
	ASA status	17 (34.69)
	ECOG	1 (2.04)

Intraoperative/Surgical	*Surgical Outcomes*	
	Compartments	39 (79.59)
	MIS; open vs. laparoscopic (vs. robotic)	7 (14.29)
	Conversion to open	6 (12.24)
	Bony resection (pelvis/sacrum)	27 (55.1)
	Use of instruments for bone stabilisation	1 (2.04)
	Met resection	5 (10.2)
	Operative time	36 (73.47)
	Blood loss	31 (63.27)
	Volume of transfusion intraop	10 (20.41)
	Urinary output intraop	1 (2.04)
	Fluid balance intraop	1 (2.04)
	Intraoperative mortality	3 (6.12)
	
	*Adjuncts to Operation*	
	IORT	8 (16.33)
	HIPC	1 (2.04)
	
	*Intraoperative Complications*	
	Major blood loss	5 (10.2)
	Sciatic nerve injury	2 (4.08)
	Ventricular fibrillation	1 (2.04)
	Iatrogenic bowel injury/hollow viscus injury	2 (4.08)

Pathological Outcomes	R0 resection	43 (87.76)
	R1 resection	42 (85.71)
	R2 resection	23 (46.94)
	Lateral surgical margins/CRM	6 (12.24)
	Tumour size	6 (12.24)
	Tumour grade	13 (26.53)
	Tumour stage	28 (57.14)
	Obstruction preoperatively	2 (4.08)
	Pathological necrosis/perforation	1 (2.04)
	Lymphovascular invasion	11 (22.45)
	Perineural invasion	5 (10.2)
	Tumour regression grade	2 (4.08)
	Quality of mesorectum	1 (2.04)

Reconstructive Outcomes	Flap reconstruction	28 (57.14)
	Perineal flap reconstruction technique	13 (26.53)
	Bladder reconstruction	20 (40.82)
	Bladder reconstruction technique	13 (26.53)
	Colonic anastomosis	12 (24.49)
	Vaginal/penile reconstruction	3 (6.12)

	*Reconstructive Complications*	
	Flap failure	5 (10.2)
	Urinary conduit complications (no leak)	4 (8.16)
	Anastomotic leak	24 (48.98)
	Necrosis of ileal conduit	1 (2.04)
		
Postoperative Outcomes	*Short-Term Complications*	
	Major complications (as defined by paper)	22 (44.9)
	Infection (unspecified)	1 (2.04)
	Foreign device infection	5 (10.2)
	Wound complications (unspecified)	1 (2.04)
	Wound infection	26 (53.06)
	Wound dehiscence	15 (30.61)
	Hernia formation	5 (10.2)
	Stoma complication	7 (14.29)
	Urinary infection	14 (28.57)
	Urinary retention/incontinence	6 (12.24)
	Ureteric leak	9 (18.37)
	Ureteric stricture	2 (4.08)
	Ileus	14 (28.57)
	Bowel obstruction	11 (22.45)
	Bowel perforation	4 (8.16)
	Intra-abdominal/pelvic sepsis	23 (46.94)
	Osteomyelitis	1 (2.04)
	Fistula formation	18 (36.73)
	Cardiac complications	7 (14.29)
	Respiratory infection	12 (24.49)
	Respiratory failure	3 (6.12)
	DVT/pulmonary embolus	7 (14.29)
	Liver failure	1 (2.04)
	Renal complications	7 (14.29)
	Neurological complications	4 (8.16)
	Multiorgan failure	2 (4.08)
	Vocal cord paresis	1 (2.04)
	Pressure sore	2 (4.08)
	Bleeding	16 (32.65)
	Reoperation	23 (46.94)
	Readmission	8 (16.33)

	*Chronic Complications*	
	Chronic pain	1 (2.04)
	Neurogenic bladder	2 (4.08)
	Urethral obstruction	3 (6.12)
	Recurrent UTIs	2 (4.08)

	*Other*	
	Hospital stay	39 (79.59)
	ICU stay	6 (12.24)
	TPN requirement	2 (4.08)
	Return of bowel function	4 (8.16)
	Postoperative mortality (within 90 days)	32 (65.31)

Patient-Reported and Functional Outcomes	QoL (instrument used)	6 (12.24)
	QoL instrument score	4 (8.16)
	Physical wellbeing	4 (8.16)
	Social/family wellbeing	1 (2.04)
	Emotional wellbeing	2 (4.08)
	Functional wellbeing	1 (2.04)
	Bowel function	1 (2.04)
	Urinary function	1 (2.04)

Survival Outcomes	Median (or mean) time of follow-up	27 (55.1)
	Recurrence overall	23 (46.94)
	Local recurrence	21 (42.86)
	Distant recurrence	19 (38.78)
	Overall survival	37 (75.51)
	Local recurrence-free survival	6 (12.24)
	Distant disease-free survival	1 (2.04)
	Disease-specific survival	1 (2.04)
	Disease-free survival	25 (51.02)

## Appendix A

PelvEx Collaborative: Salama MM, Ryan EJ, Aalbers AGJ, Abdul Aziz N, Abecasis N, Abern M, Abraham-Nordling M, Akiyoshi T, Alahmadi R, Alberda W, Albert M, Andric M, Angeles M, Angenete E, Antoniou A, Apte S, Armitage J, Auer R, Austin KK, Aytac E, Aziz O, Bacalbasa N, Baker RP, Bali M, Baransi S, Baseckas G, Bebington B, Bedford M, Bednarski BK, Beets GL, Belli F, Berg PL, Bergzoll C, Beynon J, Biondo S, Boyle K, Bordeianou L, Brecelj E, Bremers AB, Brown KGM, Brunner M, Buchwald P, Bui A, Burgess AN, Burger JWA, Burling D, Burns EM, Byrne CM, Campain N, Canda AE, Carvalhal S, Castro L, Caycedo-Marulanda A, Ceelen W, Chan KKL, Chang GJ, Charles R, Chew MH, Chok AK, Choi GS, Chong P, Christensen HK, Clark D, Clouston H, Collins D, Colquhoun AJ, Constantinides J, Corr A, Coscia M, Cosimelli M, Cotsoglou C, Coyne PE, Croner RS, Damjanovic L, Daniels IR, Davies M, Davies RJ, Delaney CP, Delisle M, Demirli S, de Wilt JHW, Denost QD, Deutsch C, Dietz D, Domingo S, Dozois EJ, Drozdov E, Duff M, Edmundson A, Egger EK, Eglinton T, Enrique-Navascues JM, Espín-Basany E, Evans MD, Eyjólfsdóttir B, Fahy M, Fearnhead NS, Ferenschild F, Fichtner-Feigl S, Fidlers T, Flatmark K, Fleming FJ, Flor-Lorente B, Folkesson J, Foskett K, Frizelle FA, Funder JA, Gallego MA, Garcia Aguilar J, García-Granero A, García-Granero E, García-Sabrido JL, Gargiulo M, Gava VG, Gentilini L, George ML, George V, Georgiou P, Ghosh A, Ghouti L, Gil-Moreno A, Giner F, Ginther N, Glover T, Glyn T, Goffredo P, Golda T, Gögenur I, Gonzalez-Argente FX, Griffiths B, Gronchi A, Grotenhuis BA, Guerra G, Gwenaël F, Harris C, Harris DA, Hagemans JAW, Hanchanale V, Harji DP, Helbren C, Helewa RM, Hellawell G, Heriot AG, Hochman D, Hohenberger W, Holm T, Holmström A, Hompes R, Hornung B, Hurton S, Hyun E, Ito M, Iversen LH, Jalbuu G, Jeri-McFarlane S, Jenkins JT, Johnstone CSH, Jourand K, Kaffenberger S, Kandaswamy GV, Kapur S, Kanemitsu Y, Kaufman M, Kazi M, Kaul S, Kelley SR, Keller DS, , Kersting S, Ketelaers SHJ, Khan MS, Khaw J, Kim H, Kim HJ, Kiran R, Koh CE, Kok NFM, Kokelaar R, Kontovounisios C, Kose F, Koutra M, Kraft M, Kristensen HØ, Kumar S, Kusters M, Lago V, Lakkis Z, Lampe B, Langheinrich MC, Larach TT, Larkin J, Larsen SG, Larson DW, Law WL, Laurberg S, Lee PJ, Limbert M, Loria A, Lydrup ML, Lyons A, Lynch AC, Lynch N, Mackintosh M, Maciel J, Malakorn S, Manfredelli S, Mann C, Mantyh C, Mathis KL, Margues CFS, Martinez A, Martling A, Meijerink WJHJ, Merchea A, Merkel S, Mehta AM, McArthur DR, McCormick JJ, McCormick P, McDermott FD, McGrath JS, McPhee A, Maciel J, Malde S, Mirnezami A, Manfredelli S, Martinez-Gomez C, Mikalauskas S, Modest DP, Monson JRT, Morton JR, Mullaney TG, Nair R, Navarro AS, Neeff H, Negoi I, Neto JWM, Nguyen B, Nielsen MB, Nieuwenhuijzen GAP, Nilsson PJ, Nordkamp S, O’Dwyer ST, Oates J, Paarnio K, Palmer G, Pappou E, Park J, Patsouras D, Paty PB, Peacock A, Pellino G, Peterson AC, Peulen HMU, Pfeffer F, Piqeur F, Pinson J, Poggioli G, Polignano F, Proud D, Quinn M, Oliver A, Quyn A, Radwan RW, Rajendran N, Rasheed S, Rasmussen PC, Rausa E, Raza I, Regenbogen SE, Reims HM, Renehan A, Rintala J, Rocha R, Rochester M, Rohila J, Rogers A, Rothbarth J, Rottoli M, Roxburgh C, Rutten HJT, Ryan ÉJ, Rydbeck D, Safar B, Sagar PM, Sahakitrungruang C, Sahai A, Saklani A, Sammour T, Sayyed R, Schizas AMP, Schwarzkopf E, Scripcariu D, Scripcariu V, Seifert G, Sekhar H, Selvasekar C, Shaban M, Shaikh I, Simianu VV, Simillis C, Simpson JAD, Skeie-Jensen T, Smart NJ, Smart P, Smith JJ, Smith TG, Sokmen S, Solbakken AM, Solomon MJ, Sørensen MM, Sorrentino L, Spasojevic M, Steele SR, Steffens D, Stitzenberg K, Stocchi L, Stylianides NA, Swartling T, Sumrien H, Sutton PA, Swartking T, Takala H, Tan EJ, Taylor C, Taylor D, Tejedor P, Tekin A, Tekkis PP, Teras J, Thanapal MR, Thaysen HV, Thorgersen EB, Thurairaja R, Tiernan J, Toh EL, Tsarkov P, Tolenaar JL, Tsukada Y, Tsukamoto S, Tuech JJ, Turner G, Turner WH, Tuynman JB, Valente M, van Ramshorst GH, Van Nieuwenhove Y, van Rees JM, Vásquez-Jiménez W, Vather R, Verhoef C, Vierimaa M, Vizzielli G, Voogt ELK, Uehara K, Wagstaff M, Wakeman C, Warrier S, Wasmuth HH, Waters P, Weber K, Weiser MR, West CT, West MA, Westney OL, Wheeler JMD, Wild J, Wilson M, Wolthuis A, Yano H, Yip B, Yip J, Yoo RN, Zappa MA, Winter DC, Kelly ME.
